# Serum Very‐Long‐Chain Saturated Fatty Acids and the Mediating Role of Systemic Immune Inflammation in Cardiovascular Disease Risk

**DOI:** 10.1002/fsn3.72573

**Published:** 2026-11-09

**Authors:** Xinyu Chen, Long Zhou, Jun Cheng

**Affiliations:** ^1^ Chongqing Medical University–University of Leicester Joint Institute Chongqing Medical University Chongqing China; ^2^ Department of Cardiology Sichuan Provincial People's Hospital, University of Electronic Science and Technology of China Chengdu China; ^3^ Institute of Cardiovascular Diseases Sichuan Provincial People's Hospital, University of Electronic Science and Technology of China Chengdu China; ^4^ Department of Vascular Surgery The First Affiliated Hospital of Chongqing Medical University Chongqing China

**Keywords:** heart failure, myocardial infarction, neutrophil, stroke, very‐long‐chain saturated fatty acids

## Abstract

Very‐long‐chain saturated fatty acids (VLCSFAs) represent a distinct class of saturated fatty acids whose cardiovascular associations remain underexplored and controversial. This study investigated the associations of serum VLCSFAs—C20:0, C22:0, C23:0, and C24:0, and their sum (total VLCSFAs)—with myocardial infarction (MI), heart failure (HF), and stroke in 3456 NHANES 2011–2014 participants. Binary logistic and restricted cubic spline regression models were employed, with mediation analysis examining neutrophil‐related inflammatory markers. With the middle tertile as the reference, participants in the lowest tertile had higher odds of MI for C20:0 (OR 2.11, 95% CI: 1.30–3.43, *p* = 0.003) and C23:0 (OR 2.55, 95% CI: 1.54–4.22, *p* < 0.001); higher odds of HF for C22:0 (OR 1.77, 95% CI: 1.08–2.90, *p* = 0.023), C24:0 (OR 2.14, 95% CI: 1.29–3.54, *p* = 0.003), and total VLCSFAs (OR 2.02, 95% CI: 1.22–3.33, *p* = 0.006); and higher odds of stroke for C24:0 (OR 1.77, 95% CI: 1.10–2.84, *p* = 0.019). Conversely, the highest tertile of C24:0 was associated with lower odds of MI (OR 0.49, 95% CI: 0.28–0.89, *p* = 0.018), whereas the highest tertiles of the remaining VLCSFAs showed no further risk reduction. SII, NER, NHR, and NLR partially mediated the associations of C23:0, C24:0, and total VLCSFAs with HF, and NER, NHR, and NLR partially mediated that of C22:0 with HF. These findings reveal novel associations of serum VLCSFAs with MI, HF, and stroke that were most evident in the lowest tertile of serum concentrations, suggesting that VLCSFA deficiency may contribute to cardiovascular risk, partly through neutrophil‐related inflammatory pathways.

## Graphical abstract

Higher serum very‐long‐chain saturated fatty acids were inversely associated with myocardial infarction, heart failure and stroke, with neutrophil‐related inflammation partly mediating the association with heart failure.

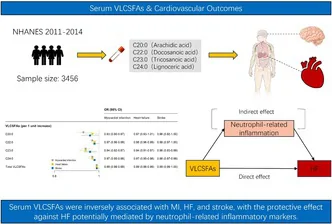

**Abbreviations**
BMI: body mass index
BP: blood pressure
CHD: coronary heart disease
CI: confidence interval
CVD: cardiovascular disease
HDL: high‐density lipoprotein
HEI: healthy eating index
HF: heart failure
LCSFAs: long‐chain saturated fatty acids
MI: myocardial infarction
NER: neutrophil‐to‐erythrocyte ratio
NHANES: National Health and Nutrition Examination Survey
NHR: neutrophil‐to‐HDL ratio
NLR: neutrophil‐to‐lymphocyte ratio
OR: odds ratio
SFAs: saturated fatty acids
SII: systemic immune inflammation index
TC: total cholesterol
VLCSFAs: very‐long‐chain saturated fatty acids

## 1 Introduction

Ischemic heart disease is expected to remain the leading cause of disability‐adjusted life‐years (DALYs) and deaths through to 2050 at the global level, while stroke is forecasted to rise from the third leading cause of global DALYs in 2022 to the second cause in 2050 (GBD 2021 Forecasting Collaborators 2024). Among the numerous risk factors for cardiovascular disease (CVD), nutrition plays a crucial role and is one of the modifiable lifestyle factors and a healthy diet is recommended as a cornerstone of CVD prevention in all individuals (Visseren et al. 2022).

For a long time, saturated fatty acids (SFAs) have been considered an important risk factor for CVD and it was strongly recommended to reduce intake of dietary SFAs and replace it with unsaturated fats to improve cardiovascular health (Sacks et al. 2017). According to the length of the carbon chain, SFAs can be categorized as either long‐chain saturated fatty acids (LCSFAs) or very‐long‐chain saturated fatty acids (VLCSFAs). SFAs with a carbon chain length of 20 or more are classified as VLCSFAs (Jiang and Sun 2022).

In recent years, several studies have observed a potential protective effect of VLCSFAs on the risk of CVD. Results from the Nurses' Health Study (NHS) and the Health Professionals Follow‐Up Study (HPFS) indicate that per 1‐standard deviation (SD) increment in total plasma VLCSFAs was linearly associated with a 10% lower coronary heart disease (CHD) risk (Malik et al. 2015). Similarly, the Guangzhou Nutrition and Health Study (GNHS) also reported that several VLCSFAs, including arachidic acid (C20:0), docosanoic acid (C22:0), and lignoceric acid (C24:0), are inversely associated with the risk of CHD in a Chinese population (Xie et al. 2024). The Cardiovascular Health Study (CHS) found that higher levels of circulating VLCSFAs are prospectively associated with a lower risk of new heart failure (HF) in US older adults (Lemaitre et al. 2018). In contrast, a case–control study nested in the Physicians' Health Study found that plasma C20:0 and C22:0 were not associated with incident HF (Matsumoto et al. 2013). Controversial results from previous studies need to be replicated in more studies. Furthermore, to our knowledge, there have been no studies specifically exploring the effect of VLCSFAs on the risk of myocardial infarction (MI) and stroke. The present study aims to investigate the effects of four types of VLCSFAs, including arachidic acid (C20:0), docosanoic acid (C22:0), tricosanoic acid (C23:0), and lignoceric acid (C24:0), on MI, HF, and stroke in the National Health and Nutrition Examination Survey (NHANES). Given the important role of neutrophils in cardiovascular inflammation and repair processes (Silvestre‐Roig et al. 2020), we will also investigate the mediating effects of several neutrophil related inflammatory markers in the association between VLCSFAs and cardiovascular outcomes.

## 2 Methods

### 2.1 Study Population

The study population was derived from the US NHANES. Briefly, NHANES is a nationwide health survey for every 2 years aimed to evaluate the health and nutritional status for US citizens of all ages. More details of the study population in the NHANES have been described elsewhere (Zhang et al. 2022). The present study selected participants from NHANES 2011–2012 and 2013–2014 because serum VLCSFAs were measured in these two cycles. In total, 19,931 participants were enrolled in NHANES 2011–2014 and we first excluded 8602 participants whose age < 20 years because the cardiovascular outcome items (MI, HF and stroke) and several covariates, including educational attainment and marital status, were collected only for adults aged 20 years and older. We then excluded 6929 participants without serum VLCSFAs data, 230 participants without other biochemistry parameters, and 714 participants with missing data on body measures. Finally, a total of 3456 individuals were included in this analysis. The procedure of the study population selection is shown in Figure 1. The NHANES 2011–2014 protocol was approved by the National Center for Health Statistics Institutional Review Board (Protocol #2011‐17) and all participants provided written informed consent.

**FIGURE 1:**
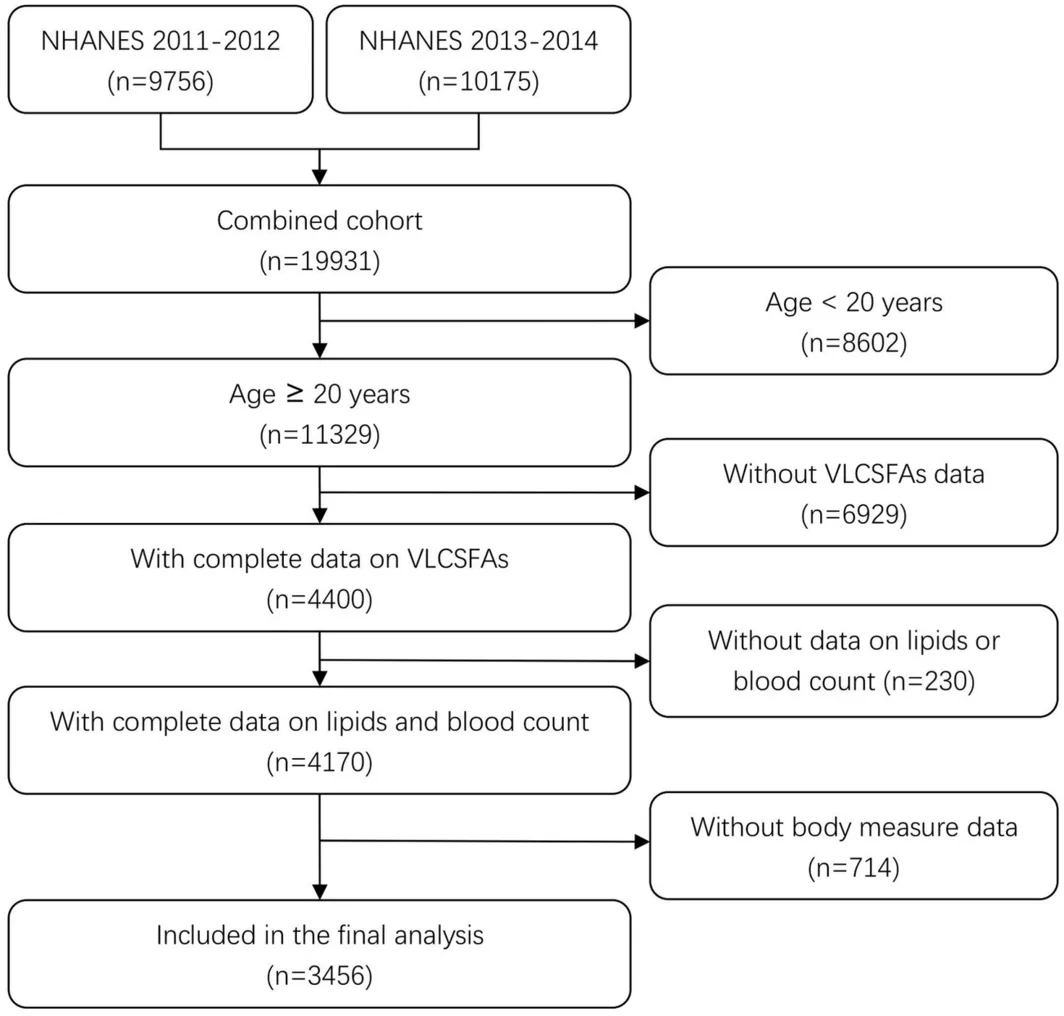
The procedure of the study population selection.

### 2.2 Exposure Measurement

The exposure variables in this study were serum arachidic acid (C20:0), docosanoic acid (C22:0), tricosanoic acid (C23:0), and lignoceric acid (C24:0). Treat serum samples with inorganic acids and bases sequentially under heating to hydrolyze triglycerides, phospholipids, and cholesterol esters into esterified fatty acids. Pentafluorobenzyl esters were formed by derivatizing the extract with pentafluorobenzyl bromide (PFBBr) when triethylamine is present. To separate the specific fatty acids of interest from other matrix components, the reaction mixture is injected onto a capillary gas chromatograph column. Fatty acids were detected using electron capture negative‐ion mass spectrometry within 34 min. Quantitation was accomplished by comparing the peak area of the analyte in the unknown with the peak area of a known amount in a calibrator solution (Tao et al. 2024).

### 2.3 Outcome Assessment

The outcomes of this study, including MI, HF, and stroke, were derived from the Medical Conditions questionnaire. Participants were asked “Has a doctor or other health professional ever told you that you had a heart attack (also called myocardial infarction)?” If someone answered “yes” to this question, they are considered to have MI. Similarly, HF and stroke were also determined in this way. This method of defining cardiovascular outcomes has been applied in many NHANES‐related studies (Abdalla et al. 2020; Brandt et al. 2022).

### 2.4 Covariates Measurement

By using the Computer‐Assisted Personal Interview (CAPI) system, trained staff collected age, sex, ethnicity, education, marital status, and family income in participants' homes. In our study, ethnicity was classified into Hispanic, White, Black, and other. The “other” category combined Non‐Hispanic Asian or American Indian or Alaska Native and Native Hawaiian or Other Pacific Islander—as well as those who reported more than one race. Education level was grouped into < high school, high school, and > high school. Marital status was combined into three groups: married/living with partner, separated/widowed/divorced, and never married. According to commonly used percentages of the poverty guidelines, family monthly poverty level index was categorized into three categories: ≤ 1.30, 1.31–1.85, and > 1.85 to reflect family income.

Body weight and standing height were measured by trained health technicians in Examination Center. Weight in kilograms divided by height in meters squared was used to compute the body mass index (BMI). Blood pressure (BP) was measured using a mercury sphygmomanometer after resting quietly in a seated position for 5 min. After determining the maximum inflation level of the sphygmomanometer's arm cuff, three consecutive blood pressure readings were taken. The average of three BP readings was used as systolic BP (SBP)/diastolic BP (DBP) in the analysis. Hypertension was defined as SBP ≥ 140 mmHg or DBP ≥ 90 mmHg or currently taking antihypertensive medications.

Smoking, drinking, sedentary time, and healthy eating index (HEI) are referred to as lifestyle factors. Smoking status was categorized into three categories: current, former, and never. We defined alcohol drinkers as having consumed alcohol at least 12 times in the previous year. Sedentary time was defined as the total time spent sitting in a normal day but excluding time spent sleeping. Two nonconsecutive 24‐h dietary recalls were conducted for all participants, and dietary data was collected. HEI was calculated based on food groups, energy, and nutrients derived from 24‐h dietary recall data. HEI consists of 13 components, including 9 adequacy components (total vegetables, greens and beans, total fruits, whole fruits, whole grains, dairy, total protein foods, seafood and plant proteins, and fatty acids) and 4 moderation components (sodium, refined grains, saturated fats, and added sugars). Each component was scored separately and added together to obtain the HEI total score (0–100) (Reedy et al. 2018).

Blood samples were collected for laboratory tests. Total cholesterol (TC) was measured on a Roche Modular P chemical analyzer by using an enzymatic method, and hypercholesterolemia was defined as TC ≥ 240 mg/dL or current cholesterol lowering medication use. High‐density lipoprotein (HDL) was measured by precipitation. Fasting glucose was measured using enzymatic method, and hemoglobin A1c was measured on a high‐performance liquid chromatography Glycohemoglobin Analyzer. Diabetes was defined as fasting glucose ≥ 126 mg/dL and/or hemoglobin A1c ≥ 6.5% and/or usage of insulin or hypoglycemic medications.

A complete blood count and a distribution of blood cells for all participants were performed on the Beckman Coulter DxH 800 instrument. Several ratios derived from neutrophil counts, including the systemic immune inflammation index (SII), the neutrophil‐to‐erythrocyte ratio (NER), the neutrophil‐to‐HDL ratio (NHR), and the neutrophil‐to‐lymphocyte ratio (NLR), were used as potential mediators in this analysis (Boughanem et al. 2025). These ratios are calculated using the following formulas:

SII=neutrophil count×platelet count/lymphocyte count

NER=neutrophil count/erythrocyte count

NHR=neutrophil count/HDL

NLR=neutrophil count/lymphocyte count

### 2.5 Statistical Analysis

In accordance with the NHANES Analytic Guidelines, the sampling weight was incorporated in the analysis. The serum fatty acid subsample weight, which was specifically constructed for participants selected into the fatty acid measurement subsample, was used as the weighting variable. Continuous variables were presented as weighted mean ± standard error (SE) and categorical variables were presented as *n* (weighted percentage). Four types of serum VLCSFAs were used in the analysis both as categorical (tertiles, with the middle tertile as the reference) and continuous (per 1 μmol/L) variables, and total VLCSFAs, defined as the sum of the four individual VLCSFAs (C20:0, C22:0, C23:0 and C24:0), were additionally analyzed in the same manner. We used the middle tertile as the reference because this category corresponded to the usual range of circulating concentrations, and it allowed the odds of each outcome to be compared in both directions from the center of the distribution. Methodological evaluations of reference‐category choice indicate that using an extreme category as the reference can reduce precision and obscure the interpretation of effect estimates (Frøslie et al. 2010). We tested differences in characteristics between groups with student *t*‐tests for continuous variables and χ^2^ tests for categorical variables. Binary logistic regression model was applied to evaluate the effects of serum VLCSFAs on MI, HF, and stroke, and multivariable adjusted odds ratio (OR) and 95% confidence interval (CI) were calculated. We included age, sex, ethnicity, education, marital status, family monthly poverty levels, smoking status, drinking status, sedentary time, healthy eating index, BMI, hypertension, diabetes, and hypercholesterolemia as covariates. Restricted cubic spline regression model was additionally used to explore the dose–response relationship between different VLCSFAs and cardiovascular outcomes. In the primary analysis, participants with missing values for any of the covariates were excluded, leaving 3456 participants for the complete‐case analysis. Because serum VLCSFA concentrations were available for all 4400 participants, we additionally performed multiple imputation by chained equations (MICE) to handle missing covariate data as a sensitivity analysis. We finally conducted a series mediation analysis to examine potential role of neutrophil related inflammatory markers in the association between VLCSFAs and cardiovascular outcomes. The variables of VLCSFAs and neutrophil related inflammatory markers were standardized before mediation analyses in order to make the results more comparable across different variables. The mediation analysis was performed using the R mediation package, which is freely available for download via the Comprehensive R Archive Network (CRAN) at http://CRAN.R‐project.org/package=mediation. A brief description of the mediation analysis has been explained elsewhere (Chen et al. 2022; Liu et al. 2022). All statistical analyses were performed using R version 4.4.3 (R Foundation for Statistical Computing, Vienna, Austria). A two‐tailed *p* < 0.05 was considered statistical significance.

## 3 Results

This study included 3456 participants (1705 men and 1751 women) with a weighted mean ± SE age of 47.3 ± 0.3 years. Table 1 summarizes the weighted characteristics of the study population overall and according to whether MI, HF, or stroke was diagnosed by a physician. Across all three outcomes, affected participants were markedly older, more likely to be White, less educated, and more often current or former smokers or living without a partner (all *p* < 0.0001); men were overrepresented in MI (62.2% vs. 48.3%) and HF (54.3% vs. 48.6%) but slightly underrepresented in stroke (45.5% vs. 48.9%). Hypertension, diabetes, and hypercholesterolemia were consistently more prevalent in all three groups (all *p* < 0.0001), whereas lifestyle factors differed by outcome: BMI and sedentary time were significantly higher only in HF, with no significant differences for MI or stroke. Most importantly, serum C22:0, C23:0, C24:0, and total VLCSFAs were significantly lower in participants with MI, HF, or stroke than in those without (all *p* < 0.05), whereas C20:0 was significantly lower only in MI (22.2 ± 0.4 vs. 23.6 ± 0.1 μmol/L, *p* = 0.0104) and did not differ significantly for HF (*p* = 0.3019) or stroke (*p* = 0.1128).

**TABLE 1:** Characteristics of the study population (*n* = 3456).

Characteristics	Overall	Myocardial infarction			Heart failure			Stroke		
	(*n* = 3456)	No (*n* = 3335)	Yes (*n* = 121)	*p*	No (*n* = 3355)	Yes (*n* = 101)	*p*	No (*n* = 3340)	Yes (*n* = 116)	*p*
Age, year	47.3 ± 0.3	46.7 ± 0.3	65.4 ± 1.0	< 0.0001	46.9 ± 0.3	63.6 ± 1.3	< 0.0001	46.8 ± 0.3	63.0 ± 1.2	< 0.0001
Men, *n* (%)	1705 (48.8)	1623 (48.3)	82 (62.2)	< 0.0001	1649 (48.6)	56 (54.3)	< 0.0001	1650 (48.9)	55 (45.5)	< 0.0001
Ethnicity, *n* (%)										
Hispanic	742 (13.9)	723 (14.1)	19 (7.1)	< 0.0001	724 (14.0)	18 (9.0)	< 0.0001	724 (14.0)	18 (7.8)	< 0.0001
White	1472 (67.7)	1400 (67.2)	72 (81.6)		1414 (67.5)	58 (76.5)		1414 (67.7)	58 (69.6)	
Black	742 (11.5)	722 (11.7)	20 (7.0)		722 (11.5)	20 (10.0)		711 (11.5)	31 (12.4)	
Others	500 (6.9)	490 (7.0)	10 (4.3)		495 (7.0)	5 (4.5)		491 (6.8)	9 (10.2)	
Education, *n* (%)										
< High school	727 (15.6)	696 (15.3)	31 (23.3)	< 0.0001	696 (15.1)	31 (32.0)	< 0.0001	689 (15.2)	38 (29.2)	< 0.0001
High school	714 (19.3)	671 (18.9)	43 (32.2)		690 (19.1)	24 (26.0)		690 (19.2)	24 (22.1)	
> High school	2015 (65.1)	1968 (65.8)	47 (44.5)		1969 (65.7)	46 (42.0)		1961 (65.6)	54 (48.7)	
Marital status, *n* (%)										
Married/Living with partner	2066 (64.0)	1995 (63.9)	71 (65.1)	< 0.0001	2011 (63.9)	55 (65.5)	< 0.0001	2001 (63.9)	65 (66.7)	< 0.0001
Separated/Widowed/Divorced	683 (16.5)	639 (16.1)	44 (30.1)		648 (16.2)	35 (28.0)		639 (16.1)	44 (29.5)	
Never married	707 (19.5)	701 (20.0)	6 (4.8)		696 (19.9)	11 (6.5)		700 (20.0)	7 (3.8)	
Family monthly poverty level, *n* (%)										
≤ 1.30	1227 (26.2)	1183 (26.1)	44 (30.2)		1183 (26.0)	44 (32.6)		1188 (26.1)	39 (31.6)	
1.31–1.85	478 (11.5)	449 (11.1)	29 (22.5)	< 0.0001	459 (11.4)	19 (16.9)	< 0.0001	451 (11.2)	27 (20.7)	< 0.0001
> 1.85	1751 (62.3)	1703 (62.8)	48 (47.3)		1713 (62.6)	38 (50.5)		1701 (62.7)	50 (47.7)	
Smoking status, *n* (%)										
Current	681 (19.3)	653 (19.0)	28 (26.3)	< 0.0001	660 (19.0)	21 (28.4)	< 0.0001	650 (18.9)	31 (30.5)	< 0.0001
Former	791 (23.8)	735 (23.0)	56 (44.9)		751 (23.2)	40 (43.4)		751 (23.4)	40 (37.2)	
Never	1984 (57.0)	1947 (58.0)	37 (28.8)		1944 (57.8)	40 (28.2)		1939 (57.7)	45 (32.3)	
Alcohol drinker, *n* (%)	2463 (77.5)	2370 (77.4)	93 (79.8)	< 0.0001	2388 (77.4)	75 (80.7)	< 0.0001	2384 (77.4)	79 (80.0)	< 0.0001
Sedentary time, hour/day	6.6 ± 0.1	6.6 ± 0.1	6.9 ± 0.3	0.3421	6.6 ± 0.1	7.5 ± 0.4	0.0071	6.6 ± 0.1	7.2 ± 0.3	0.0549
Healthy eating index	54.4 ± 0.2	54.4 ± 0.2	53.6 ± 1.2	0.5582	54.4 ± 0.2	53.0 ± 1.2	0.3374	54.3 ± 0.2	54.5 ± 1.2	0.8830
BMI, kg/m^2^	29.0 ± 0.1	29.0 ± 0.1	29.5 ± 0.6	0.3971	28.9 ± 0.1	31.3 ± 0.8	0.0015	29.0 ± 0.1	29.6 ± 0.8	0.3391
Hypertension, *n* (%)	1221 (32.4)	1133 (31.1)	88 (70.4)	< 0.0001	1142 (31.3)	79 (76.4)	< 0.0001	1134 (31.2)	87 (71.5)	< 0.0001
Diabetes, *n* (%)	555 (12.0)	500 (11.0)	55 (43.2)	< 0.0001	515 (11.4)	40 (36.3)	< 0.0001	516 (11.5)	39 (29.5)	< 0.0001
Hypercholesterolemia, *n* (%)	1006 (29.3)	919 (27.7)	87 (76.6)	< 0.0001	946 (28.6)	60 (56.8)	< 0.0001	946 (28.5)	60 (56.2)	< 0.0001
Serum VLCSFAs										
Arachidic acid (C20:0), μmol/L	23.5 ± 0.1	23.6 ± 0.1	22.2 ± 0.4	0.0104	23.5 ± 0.1	22.9 ± 0.5	0.3019	23.6 ± 0.1	22.7 ± 0.5	0.1128
Docosanoic acid (C22:0), μmol/L	68.5 ± 0.3	68.8 ± 0.3	60.8 ± 1.4	< 0.0001	68.6 ± 0.3	62.9 ± 1.9	0.0020	68.6 ± 0.3	64.9 ± 1.6	0.0303
Tricosanoic acid (C23:0), μmol/L	29.3 ± 0.1	29.4 ± 0.1	26.6 ± 0.7	0.0002	29.3 ± 0.1	27.1 ± 0.7	0.0090	29.3 ± 0.1	27.6 ± 0.7	0.0248
Lignoceric acid (C24:0), μmol/L	58.2 ± 0.2	58.5 ± 0.3	50.6 ± 1.2	< 0.0001	58.4 ± 0.2	51.9 ± 1.5	< 0.0001	58.4 ± 0.3	54.3 ± 1.3	0.0051
Total VLCSFAs, μmol/L	179.6 ± 0.7	180.2 ± 0.7	160.3 ± 3.5	< 0.0001	179.9 ± 0.7	164.9 ± 4.4	0.0008	179.9 ± 0.7	169.4 ± 3.8	0.0128

*Note:* Continuous variables are presented as weighted mean ± standard error, and categorical variables are presented as number (weighted percentage). Total VLCSFAs: sum of C20:0, C22:0, C23:0, and C24:0.

Abbreviations: BMI, body mass index; VLCSFAs, very‐long‐chain saturated fatty acids.

The associations of serum VLCSFAs with MI, HF, and stroke are shown in Table 2, with the middle tertile as the reference group. After multivariable adjustment (Model 2), participants in the lowest tertile had significantly higher odds of MI for C20:0 (OR 2.11, 95% CI: 1.30–3.43, *p* = 0.003) and C23:0 (OR 2.55, 95% CI: 1.54–4.22, *p* < 0.001); higher odds of HF for C22:0 (OR 1.77, 95% CI: 1.08–2.90, *p* = 0.023), C24:0 (OR 2.14, 95% CI: 1.29–3.54, *p* = 0.003), and total VLCSFAs (OR 2.02, 95% CI: 1.22–3.33, *p* = 0.006); and higher odds of stroke for C24:0 (OR 1.77, 95% CI: 1.10–2.84, *p* = 0.019). The highest tertile of C24:0 was associated with significantly lower odds of MI compared with the middle tertile (OR 0.49, 95% CI: 0.28–0.89, *p* = 0.018), while no significant differences were observed between the highest and middle tertiles of other VLCSFAs for all three outcomes (all *p* > 0.05). In continuous analyses per 1 μmol/L increase, higher serum concentrations of all VLCSFAs except C20:0 for HF were significantly inversely associated with risks of MI, HF, and stroke after full adjustment.

**TABLE 2:** ORs (95% CIs) for the associations of serum very‐long‐chain saturated fatty acids (VLCSFAs) with myocardial infarction, heart failure, and stroke.

Fatty acid	Myocardial infarction			Heart failure			Stroke		
	Case No. (%)	Model 1	Model 2	Case No. (%)	Model 1	Model 2	Case No. (%)	Model 1	Model 2
C20:0 (Arachidic acid)									
T1 (lowest: < 20.8)	62 (5.4)	2.20 (1.38–3.48), *p* < 0.001	2.11 (1.30–3.43), *p* = 0.003	45 (3.9)	1.53 (0.95–2.46), *p* = 0.078	1.37 (0.84–2.23), *p* = 0.205	44 (3.9)	1.17 (0.75–1.82), *p* = 0.492	1.07 (0.68–1.69), *p* = 0.769
T2 (Ref.: 20.8 ~ 25.1)	30 (2.6)	1 (Ref.)	1 (Ref.)	32 (2.8)	1 (Ref.)	1 (Ref.)	42 (3.6)	1 (Ref.)	1 (Ref.)
T3 (highest: ≥ 25.2)	29 (2.5)	1.03 (0.60–1.76), *p* = 0.916	0.91 (0.52–1.60), *p* = 0.744	24 (2.1)	0.68 (0.39–1.19), *p* = 0.176	0.68 (0.39–1.21), *p* = 0.188	30 (2.6)	0.60 (0.37–0.99), *p* = 0.044	0.61 (0.36–1.01), *p* = 0.053
Continuous (per 1 μmol/L)	—	0.94 (0.90–0.98), *p* = 0.001	0.93 (0.90–0.97), *P* = 0.001	—	0.96 (0.92–1.00), *p* = 0.048	0.97 (0.93–1.01), *p* = 0.140	—	0.95 (0.92–0.99), *p* = 0.007	0.96 (0.92–1.00), *p* = 0.033
C22:0 (Docosanoic acid)									
T1 (lowest: < 59.2)	60 (5.3)	1.56 (1.00–2.44), *p* = 0.049	1.25 (0.78–2.00), *p* = 0.358	55 (4.8)	2.01 (1.25–3.24), *p* = 0.004	1.77 (1.08–2.90), *p* = 0.023	53 (4.6)	1.75 (1.10–2.77), *p* = 0.017	1.59 (0.99–2.55), *p* = 0.056
T2 (Ref.: 59.2 ~ 72.0)	35 (3.0)	1 (Ref.)	1 (Ref.)	28 (2.4)	1 (Ref.)	1 (Ref.)	32 (2.8)	1 (Ref.)	1 (Ref.)
T3 (highest: ≥ 72.1)	26 (2.3)	0.75 (0.44–1.27), *p* = 0.278	0.62 (0.36–1.08), *p* = 0.092	18 (1.6)	0.59 (0.32–1.08), *p* = 0.086	0.59 (0.32–1.10), *p* = 0.100	31 (2.7)	0.87 (0.52–1.45), *p* = 0.593	0.90 (0.53–1.52), *p* = 0.693
Continuous (per 1 μmol/L)	—	0.97 (0.96–0.99), *p* < 0.001	0.97 (0.96–0.99), *P* < 0.001	—	0.97 (0.96–0.99), *P* < 0.001	0.98 (0.96–0.99), *P* < 0.001	—	0.98 (0.97–0.99), *p* = 0.002	0.98 (0.97–1.00), *p* = 0.014
C23:0 (Tricosanoic acid)									
T1 (lowest: < 25.2)	67 (5.9)	2.65 (1.64–4.28), *P* < 0.001	2.55 (1.54–4.22), *P* < 0.001	48 (4.2)	1.73 (1.07–2.81), *p* = 0.025	1.54 (0.94–2.53), *p* = 0.089	48 (4.2)	1.44 (0.91–2.26), *p* = 0.117	1.33 (0.84–2.12), *p* = 0.223
T2 (Ref.: 25.2 ~ 31.2)	26 (2.2)	1 (Ref.)	1 (Ref.)	30 (2.6)	1 (Ref.)	1 (Ref.)	38 (3.3)	1 (Ref.)	1 (Ref.)
T3 (highest: ≥ 31.3)	28 (2.4)	1.12 (0.64–1.95), *p* = 0.700	1.13 (0.64–2.02), *p* = 0.668	23 (2.0)	0.69 (0.39–1.22), *p* = 0.201	0.75 (0.42–1.33), *p* = 0.327	30 (2.6)	0.67 (0.41–1.11), *p* = 0.119	0.74 (0.44–1.24), *p* = 0.255
Continuous (per 1 μmol/L)	—	0.94 (0.91–0.97), *p* < 0.001	0.94 (0.92–0.97), *P* < 0.001	—	0.93 (0.90–0.96), *P* < 0.001	0.94 (0.91–0.97), *P* < 0.001	—	0.95 (0.92–0.97), *P* < 0.001	0.96 (0.93–0.99), *p* = 0.003
C24:0 (Lignoceric acid)									
T1 (lowest: < 50.4)	65 (5.7)	1.74 (1.12–2.69), *p* = 0.013	1.42 (0.90–2.25), *p* = 0.133	60 (5.3)	2.52 (1.54–4.11), *p* < 0.001	2.14 (1.29–3.54), *p* = 0.003	57 (5.0)	1.93 (1.22–3.06), *p* = 0.005	1.77 (1.10–2.84), *p* = 0.019
T2 (Ref.: 50.4 ~ 61.7)	35 (3.0)	1 (Ref.)	1 (Ref.)	24 (2.1)	1 (Ref.)	1 (Ref.)	30 (2.6)	1 (Ref.)	1 (Ref.)
T3 (highest: ≥ 61.8)	21 (1.8)	0.59 (0.34–1.03), *p* = 0.063	0.49 (0.28–0.89), *p* = 0.018	17 (1.5)	0.67 (0.36–1.27), *p* = 0.219	0.70 (0.37–1.34), *p* = 0.284	29 (2.5)	0.91 (0.54–1.53), *p* = 0.717	0.94 (0.55–1.61), *p* = 0.811
Continuous (per 1 μmol/L)	—	0.97 (0.95–0.98), *p* < 0.001	0.97 (0.95–0.98), *P* < 0.001	—	0.96 (0.94–0.97), *P* < 0.001	0.97 (0.95–0.98), *P* < 0.001	—	0.98 (0.96–0.99), *P* < 0.001	0.98 (0.97–0.99), *p* = 0.005
Total VLCSFAs									
T1 (lowest: < 157.0)	64 (5.6)	1.93 (1.23–3.02), *p* = 0.004	1.62 (1.00–2.61), *p* = 0.049	57 (5.0)	2.32 (1.43–3.77), *p* < 0.001	2.02 (1.22–3.33), *p* = 0.006	54 (4.7)	1.66 (1.06–2.60), *p* = 0.026	1.55 (0.98–2.46), *p* = 0.064
T2 (Ref.: 157.0 ~ 188.9)	32 (2.7)	1 (Ref.)	1 (Ref.)	26 (2.2)	1 (Ref.)	1 (Ref.)	35 (3.0)	1 (Ref.)	1 (Ref.)
T3 (highest: ≥ 189.0)	25 (2.2)	0.78 (0.45–1.35), *p* = 0.374	0.66 (0.38–1.17), *p* = 0.159	18 (1.6)	0.63 (0.34–1.16), *p* = 0.137	0.65 (0.35–1.22), *p* = 0.181	27 (2.3)	0.67 (0.40–1.13), *p* = 0.134	0.72 (0.42–1.22), *p* = 0.221
Continuous (per 1 μmol/L)	—	0.99 (0.98–0.99), *p* < 0.001	0.99 (0.98–0.99), *P* < 0.001	—	0.99 (0.98–0.99), *P* < 0.001	0.99 (0.98–0.99), *P* < 0.001	—	0.99 (0.99–1.00), *P* < 0.001	0.99 (0.99–1.00), *p* = 0.005

*Note:* Model 1: Adjusted for age and sex. Model 2: Model 1 plus ethnicity, education, marital status, family monthly poverty levels, smoking status, drinking status, sedentary time, healthy eating index, body mass index, hypertension, diabetes, and hypercholesterolemia. Tertile 2 was the reference group.

Abbreviations: CI, confidence interval; OR, odds ratio.

In a sensitivity analysis restricted to the 4400 participants with complete serum VLCSFA measurements, in which missing covariates were imputed using MICE, the associations of VLCSFAs with MI, HF, and stroke were consistent with those observed in the primary complete‐case analysis, with similar effect estimates and confidence intervals (Table S1), indicating that the findings were robust to the handling of missing data.

The dose–response associations of serum VLCSFAs with MI, HF, and stroke are shown in Figure 2. Serum C20:0, C22:0, C23:0, C24:0, and total VLCSFAs were inversely associated with MI in linear shapes. Serum C23:0, C24:0, and total VLCSFAs were inversely and linearly associated with HF. There is a nonlinear association between serum C22:0 and HF (*p* for nonlinear = 0.036). Serum C20:0 did not significantly associate with either stroke or HF. Serum C22:0, C23:0, C24:0, and total VLCSFAs were found to be linearly and inversely associated with stroke.

**FIGURE 2:**
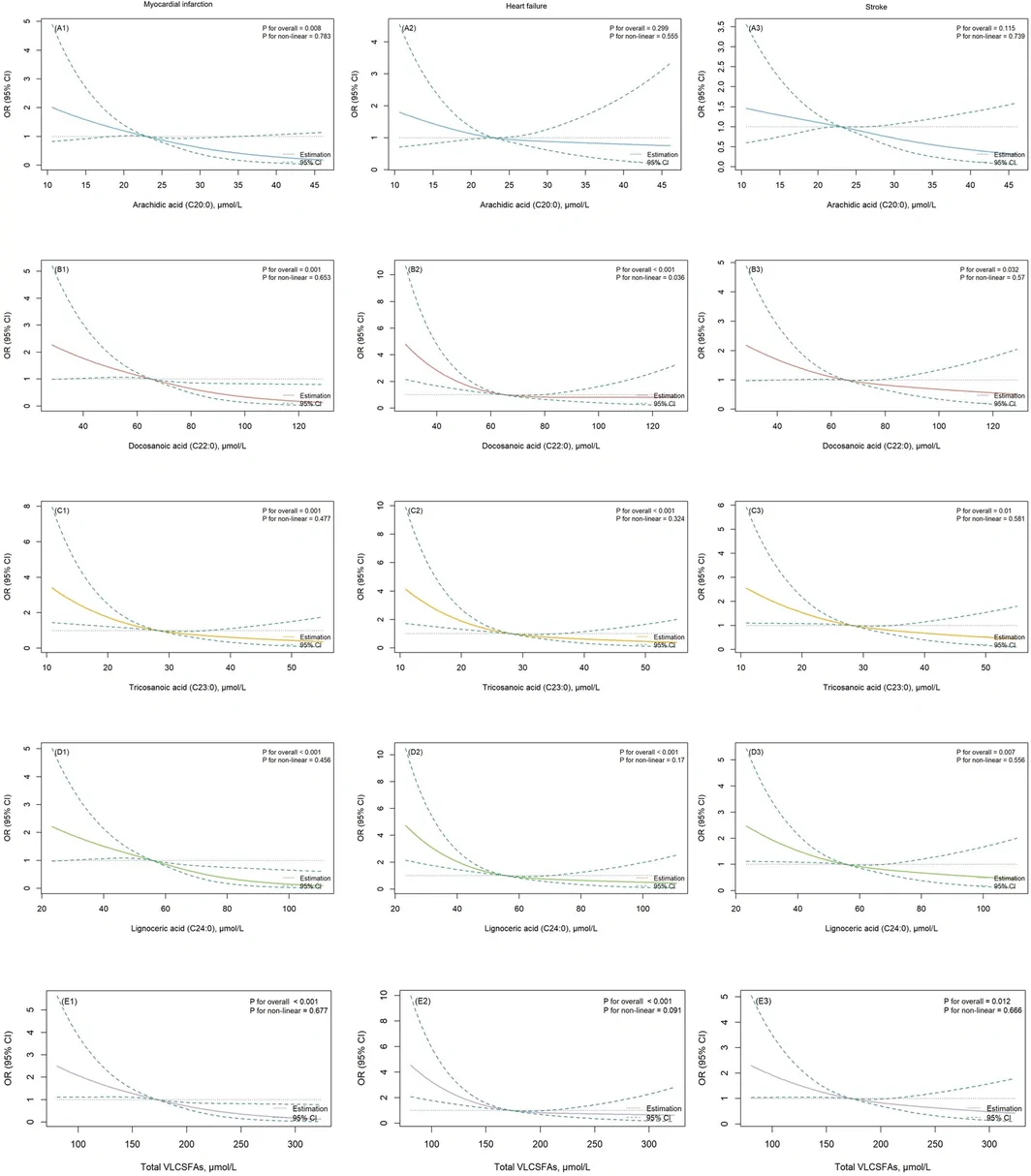
Dose–response associations of serum very‐long‐chain saturated fatty acids (VLCSFAs) with myocardial infarction (MI), heart failure (HF), and stroke. Restricted cubic spline model was adjusted for age, sex, ethnicity, education, marital status, family monthly poverty levels, smoking status, drinking status, sedentary time, healthy eating index, body mass index, hypertension, diabetes, and hypercholesterolemia. CI, confidence interval; OR, odds ratio. Panel A1, A2, and A3 represent dose–response association of serum C20:0 with MI, HF, and stroke, respectively. Panel B1, B2, and B3 represent dose–response association of serum C22:0 with MI, HF, and stroke, respectively. Panel C1, C2, and C3 represent dose–response association of serum C23:0 with MI, HF, and stroke, respectively. Panel D1, D2, and D3 represent dose–response association of serum C24:0 with MI, HF, and stroke, respectively. Panel E1, E2, and E3 represent dose–response association of total VLCSFAs with MI, HF, and stroke, respectively.

Results from mediation analysis for the role of neutrophil related inflammatory markers in the association between VLCSFAs and cardiovascular outcomes are shown in Table 3. The associations of serum C20:0 with MI, HF and stroke were not mediated by SII, NER, NHR or NLR. Likewise, there was no evidence that SII, NER, NHR or NLR mediated the associations of serum C22:0, C23:0 and C24:0, or of total VLCSFAs, with MI or stroke. For HF, the association with C22:0 was partially mediated by NER, NHR and NLR (proportion mediated 3.0%–4.4%), and the associations with C23:0, C24:0 and total VLCSFAs by SII, NER, NHR and NLR (1.5%–5.5%).

**TABLE 3:** Mediation effects of neutrophil related inflammatory markers in the association between VLCSFAs and cardiovascular outcomes.

Exposure and outcome	Mediator	Total effect	Direct effect	Indirect effect	Mediated proportion, %
Arachidic acid (C20:0)					
Myocardial infarction	SII	−0.3595 (−0.6210 to −0.1460)***	−0.3580 (−0.6220 to −0.1479)***	−0.0005 (−0.0062 to 0.0071)	…
	NER	−0.3595 (−0.6155 to −0.1522)***	−0.3494 (−0.6106 to −0.1395)**	−0.0023 (−0.0096 to 0.0031)	…
	NHR	−0.3595 (−0.6140 to −0.1630)**	−0.3409 (−0.5906 to −0.1505)**	−0.0055 (−0.0156 to 0.0009)	…
	NLR	−0.3595 (−0.6183 to −0.1427)**	−0.3570 (−0.6182 to −0.1428)***	−0.0011 (−0.0123 to 0.0093)	…
Heart failure	SII	−0.1579 (−0.4173 to 0.0594)	−0.1391 (−0.4003 to 0.0789)	−0.0048 (−0.0128 to 0.0018)	…
	NER	−0.1579 (−0.4211 to 0.0785)	−0.1306 (−0.3944 to 0.1027)	−0.0044 (−0.0149 to 0.0056)	…
	NHR	−0.1579 (−0.4286 to 0.0706)	−0.1277 (−0.3967 to 0.0970)	−0.0091 (−0.0228 to −0.0001)*	…
	NLR	−0.1579 (−0.4280 to 0.0669)	−0.1210 (−0.3927 to 0.1002)	−0.0154 (−0.0328 to −0.0048)**	…
Stroke	SII	−0.2270 (−0.4497 to −0.0407)*	−0.2117 (−0.4312 to −0.0345)*	−0.0039 (−0.0122 to 0.0019)	…
	NER	−0.2270 (−0.4506 to −0.0323)*	−0.2150 (−0.4350 to −0.0231)*	−0.0022 (−0.0088 to 0.0030)	…
	NHR	−0.2270 (−0.4490 to −0.0293)*	−0.2173 (−0.4381 to −0.0177)*	−0.0034 (−0.0126 to 0.0033)	…
	NLR	−0.2270 (−0.4517 to −0.0297)*	−0.2158 (−0.4436 to −0.0219)*	−0.0050 (−0.0164 to 0.0040)	…
Docosanoic acid (C22:0)					
Myocardial infarction	SII	−0.4482 (−0.7156 to −0.2529)***	−0.4471 (−0.7172 to −0.2530)***	−0.0004 (−0.0055 to 0.0055)	…
	NER	−0.4482 (−0.7057 to −0.2357)***	−0.4368 (−0.6996 to −0.2277)***	−0.0067 (−0.0187 to 0.0041)	…
	NHR	−0.4482 (−0.7091 to −0.2295)***	−0.4273 (−0.6765 to −0.2089)***	−0.0103 (−0.0259 to 0.0044)	…
	NLR	−0.4482 (−0.7165 to −0.2190)***	−0.4466 (−0.7183 to −0.2219)***	−0.0008 (−0.0172 to 0.0125)	…
Heart failure	SII	−0.4171 (−0.7444 to −0.1448)**	−0.4066 (−0.7400 to −0.1384)**	−0.0039 (−0.0121 to 0.0025)	…
	NER	−0.4171 (−0.7750 to −0.1469)***	−0.3914 (−0.7525 to −0.1163)**	−0.0127 (−0.0262 to −0.0028)*	3.0 (0.5 to 10.2)*
	NHR	−0.4171 (−0.7515 to −0.1559)***	−0.3831 (−0.7285 to −0.1277)**	−0.0176 (−0.0341 to −0.0055)**	4.2 (1.1 to 14.0)**
	NLR	−0.4171 (−0.7759 to −0.1276)**	−0.3796 (−0.7481 to −0.0929)*	−0.0182 (−0.0394 to −0.0050)***	4.4 (0.9 to 16.1)**
Stroke	SII	−0.2694 (−0.5156 to −0.0569)*	−0.2585 (−0.5083 to −0.0445)*	−0.0033 (−0.0116 to 0.0017)	…
	NER	−0.2694 (−0.5082 to −0.0347)*	−0.2577 (−0.4999 to −0.0157)*	−0.0066 (−0.0167 to 0.0018)	…
	NHR	−0.2694 (−0.5410 to −0.0648)*	−0.2584 (−0.5282 to −0.0559)*	−0.0065 (−0.0201 to 0.0067)	…
	NLR	−0.2694 (−0.5116 to −0.0496)*	−0.2580 (−0.5051 to −0.0381)*	−0.0062 (−0.0204 to 0.0044)	…
Tricosanoic acid (C23:0)					
Myocardial infarction	SII	−0.4337 (−0.6965 to −0.1972)***	−0.4326 (−0.7017 to −0.1963)***	−0.0006 (−0.0125 to 0.0148)	…
	NER	−0.4337 (−0.7179 to −0.1832)***	−0.4218 (−0.7032 to −0.1733)***	−0.0103 (−0.0278 to 0.0045)	…
	NHR	−0.4337 (−0.7376 to −0.2088)***	−0.4106 (−0.7177 to −0.1789)**	−0.0163 (−0.0395 to 0.0063)	…
	NLR	−0.4337 (−0.7296 to −0.2029)***	−0.4319 (−0.7321 to −0.1978)***	−0.0011 (−0.0201 to 0.0166)	…
Heart failure	SII	−0.4792 (−0.7642 to −0.2274)**	−0.4507 (−0.7443 to −0.1968)**	−0.0095 (−0.0245 to −0.0007)*	2.0 (0.1 to 6.9)*
	NER	−0.4792 (−0.7635 to −0.2046)***	−0.4442 (−0.7405 to −0.1626)***	−0.0184 (−0.0345 to −0.0054)*	3.8 (1.0 to 11.1)*
	NHR	−0.4792 (−0.7938 to −0.2301)***	−0.4352 (−0.7566 to −0.1784)***	−0.0259 (−0.0507 to −0.0060)*	5.4 (1.0 to 15.0)*
	NLR	−0.4792 (−0.7873 to −0.2172)***	−0.4269 (−0.7359 to −0.1583)***	−0.0229 (−0.0515 to −0.0056)**	4.8 (1.1 to 15.0)**
Stroke	SII	−0.3306 (−0.5931 to −0.1126)**	−0.3075 (−0.5597 to −0.0876)*	−0.0083 (−0.0225 to 0.0018)	…
	NER	−0.3306 (−0.5788 to −0.0905)**	−0.3141 (−0.5719 to −0.0768)*	−0.0090 (−0.0228 to 0.0036)	…
	NHR	−0.3306 (−0.5776 to −0.1000)**	−0.3176 (−0.5643 to −0.0944)**	−0.0084 (−0.0318 to 0.0138)	…
	NLR	−0.3306 (−0.6107 to −0.1070)**	−0.3160 (−0.5923 to −0.0846)*	−0.0072 (−0.0263 to 0.0069)	…
Lignoceric acid (C24:0)					
Myocardial infarction	SII	−0.4690 (−0.7075 to −0.2526)***	−0.4681 (−0.7055 to −0.2466)***	−0.0008 (−0.0111 to 0.0096)	…
	NER	−0.4690 (−0.6972 to −0.2785)***	−0.4573 (−0.6854 to −0.2682)***	−0.0100 (−0.0264 to 0.0051)	…
	NHR	−0.4690 (−0.6994 to −0.2603)***	−0.4451 (−0.6802 to −0.2316)***	−0.0151 (−0.0396 to 0.0098)	…
	NLR	−0.4690 (−0.7080 to −0.2633)***	−0.4679 (−0.7070 to −0.2571)***	−0.0008 (−0.0195 to 0.0161)	…
Heart failure	SII	−0.5127 (−0.7974 to −0.2785)***	−0.5008 (−0.7903 to −0.2636)***	−0.0081 (−0.0184 to −0.0013)*	1.6 (0.2 to 4.7)*
	NER	−0.5127 (−0.7984 to −0.2673)***	−0.4841 (−0.7848 to −0.2398)***	−0.0189 (−0.0355 to −0.0042)*	3.7 (0.7 to 9.5)*
	NHR	−0.5127 (−0.8060 to −0.2846)***	−0.4666 (−0.7685 to −0.2307)***	−0.0282 (−0.0551 to −0.0041)*	5.5 (0.8 to 14.1)*
	NLR	−0.5127 (−0.7982 to −0.2360)***	−0.4765 (−0.7575 to −0.1977)**	−0.0222 (−0.0498 to −0.0066)***	4.3 (1.0 to 13.8)***
Stroke	SII	−0.3202 (−0.5768 to −0.1188)***	−0.3085 (−0.5686 to −0.1065)**	−0.0070 (−0.0181 to 0.0011)	…
	NER	−0.3202 (−0.5734 to −0.1041)**	−0.3078 (−0.5539 to −0.0969)**	−0.0099 (−0.0235 to 0.0037)	…
	NHR	−0.3202 (−0.5614 to −0.1050)**	−0.3076 (−0.5468 to −0.0878)**	−0.0088 (−0.0351 to 0.0176)	…
	NLR	−0.3202 (−0.5538 to −0.1135)**	−0.3094 (−0.5446 to −0.1000)**	−0.0075 (−0.0266 to 0.0079)	…
Total VLCSFAs					
Myocardial infarction	SII	−0.4768 (−0.7116 to −0.2709)***	−0.4758 (−0.7116 to −0.2681)***	−0.0006 (−0.0091 to 0.0079)	…
	NER	−0.4768 (−0.7163 to −0.2411)***	−0.4650 (−0.7027 to −0.2370)***	−0.0082 (−0.0220 to 0.0052)	…
	NHR	−0.4768 (−0.7159 to −0.2868)***	−0.4538 (−0.6965 to −0.2670)***	−0.0129 (−0.0328 to 0.0098)	…
	NLR	−0.4768 (−0.7409 to −0.2673)***	−0.4758 (−0.7404 to −0.2592)***	−0.0006 (−0.0186 to 0.0150)	…
Heart failure	SII	−0.4545 (−0.7393 to −0.1950)**	−0.4388 (−0.7256 to −0.1816)**	−0.0068 (−0.0169 to −0.0001)*	1.5 (0.0 to 5.1)*
	NER	−0.4545 (−0.7616 to −0.1972)***	−0.4251 (−0.7266 to −0.1708)***	−0.0157 (−0.0295 to −0.0036)**	3.5 (0.7 to 11.1)**
	NHR	−0.4545 (−0.7612 to −0.1712)**	−0.4129 (−0.7292 to −0.1233)**	−0.0233 (−0.0441 to −0.0044)*	5.1 (0.7 to 16.6)*
	NLR	−0.4545 (−0.7645 to −0.2085)***	−0.4129 (−0.7244 to −0.1579)**	−0.0212 (−0.0477 to −0.0062)**	4.7 (1.1 to 14.8)**
Stroke	SII	−0.3136 (−0.5648 to −0.1036)**	−0.2991 (−0.5521 to −0.0892)**	−0.0058 (−0.0162 to 0.0014)	…
	NER	−0.3136 (−0.5564 to −0.0993)**	−0.3001 (−0.5455 to −0.0786)**	−0.0080 (−0.0197 to 0.0035)	…
	NHR	−0.3136 (−0.5564 to −0.0975)**	−0.3012 (−0.5409 to −0.0803)**	−0.0077 (−0.0265 to 0.0111)	…
	NLR	−0.3136 (−0.5788 to −0.0899)**	−0.3014 (−0.5642 to −0.0726)*	−0.0069 (−0.0231 to 0.0077)	…

*Note:* Models were adjusted for age, sex, ethnicity, education, marital status, family monthly poverty levels, smoking status, sedentary time, healthy eating index, body mass index, hypertension, diabetes, and hypercholesterolemia. Effect estimates are log‐odds ratios per 1‐SD increase in VLCSFA. Bootstrap 95% confidence intervals (1000 resamples) are shown in parentheses. “…” indicates the mediated proportion was not statistically significant or not estimable.

Abbreviations: NER, neutrophil‐to‐erythrocyte ratio; NHR, neutrophil‐to‐HDL ratio; NLR, neutrophil‐to‐lymphocyte ratio; SII, systemic immune‐inflammation index; VLCSFAs, very long‐chain saturated fatty acids.

* *p* < 0.05.

** *p* < 0.01.

*** *p* < 0.001.

## 4 Discussion

In this community‐based cross‐sectional study conducted in US adults, compared with the middle tertile, the lowest serum concentrations of VLCSFAs—including arachidic acid (C20:0), docosanoic acid (C22:0), tricosanoic acid (C23:0), lignoceric acid (C24:0), and total VLCSFAs—were associated with higher odds of major cardiovascular outcomes, including MI, HF, and stroke, whereas only the highest concentrations of C24:0 were associated with lower odds of MI, with no comparable additional risk reduction for the other VLCSFAs. Additionally, we found that the relationship between serum VLCSFAs, particularly C22:0, C23:0, and C24:0, and HF may be mediated by neutrophil‐related inflammatory markers, such as SII, NER, NHR, and NLR.

SFAs are actually a general designation for a broad range of fatty acids that do not include unsaturated double bonds in the carbon chain, despite the fact that they have long been thought to be harmful to cardiovascular health. The sources, chemical structures, processes of metabolism, and biological functions for different SFA types vary greatly (Legrand and Rioux 2010). The cardiometabolic effects of SFAs differ according to chain length, and the long‐chain species (LCSFAs) have been studied far more extensively than the VLCSFAs examined here. Myristic acid (C14:0), palmitic acid (C16:0), and stearic acid (C18:0) are the predominant circulating LCSFAs, and higher concentrations of these LCSFAs have been consistently associated with an increased risk of cardiovascular disease. In the Atherosclerosis Risk in Communities (ARIC) Study, C18:0 was positively associated with incident coronary heart disease (Wang et al. 2003), and C14:0 was positively associated with incident HF (Yamagishi et al. 2008). In the Physicians' Health Study, each standard‐deviation higher level of plasma C16:0 was associated with an OR of 1.20 (95% CI: 1.04–1.38) for incident HF (Matsumoto et al. 2013), and serial biomarker measurements in the Cardiovascular Health Study showed that long‐term elevations in C16:0 were prospectively associated with a higher risk of HF (Lee et al. 2020). Collectively, these findings are consistent with meta‐analytic evidence showing that the excess risk associated with total SFA is largely attributable to C14:0 and C16:0 (Li et al. 2022), and with individual‐level analyses of 172,891 participants showing positive associations of even‐chain LCSFAs with coronary heart disease and stroke (Shi et al. 2025). Because the LCSFA–cardiovascular relationship is relatively well established and consistent, we focused on the less well characterized VLCSFAs.

Serum VLCSFAs originate both from the diet and endogenous metabolism. Some nuts, seeds, and their oils contain low amounts of VLCSFAs (Lemaitre and King 2022). VLCSFAs can also be synthesized in vivo based on LCSFAs such as C18:0 under the action of several elongases in the elongation of very long chain fatty acids (ELOVL) family of enzymes (Sassa and Kihara 2014). VLCSFAs have been reported to be associated with reduced risk of several cardiovascular outcomes and mortality in several previous studies. The CHS cohort first reported an inverse relationship between circulating VLCSFAs (C20:0, C22:0, and C24:0) and incident atrial fibrillation in US older adults (Fretts et al. 2014). Subsequently, the NHS and HPFS cohorts found that compared to individuals in the lowest quintile of total VLCSFAs, those in the highest quintile had 52% (95% CI: 28%–68%) lower risk of CHD in US men and women (Malik et al. 2015). The GNHS cohort conducted in the Chinese population replicated results of NHS and HPFS. The GNHS cohort reported that the multivariable‐adjusted hazard ratios (HRs) and 95% CIs for CHD incidence in highest vs. lowest quartiles were 0.87 (0.61–1.25) for C20:0, 0.63 (0.42–0.96) for C22:0, and 0.59 (0.41–0.85) for C24:0 (Xie et al. 2024). The current research results on the relationship between VLCSFAs and HF are inconsistent. Results from the CHS cohort showed that the HR comparing the highest quintile to the lowest quintile of VLCSFAs was 0.72 (95% CI: 0.59–0.88) for C20:0, 0.72 (95% CI: 0.60–0.87) for C22:0, and 0.67 (95% CI: 0.55–0.81) for C24:0 (Lemaitre et al. 2018). However, a nested case–control study conducted in US male physicians did not observe significant associations between VLCSFAs (C20:0 or C22:0) and HF risk (Matsumoto et al. 2013). A recent study analyzed NHANES data and found that individuals with higher circulating C22:0 and C24:0 reduced the risks of death from all‐cause and CVD (Tao et al. 2024). The CHS cohort and the Framingham Offspring cohort also confirmed the beneficial effect of VLCSFAs on mortality risk (Fretts et al. 2016; McBurney et al. 2021). It should be noted that each of these studies has some limitations that may affect the representativeness of the results. For example, the CHS cohort was conducted among older adults, the population at highest risk of HF, and results may not be generalizable to younger populations. The study population in the NHS and HPFS cohorts were white healthcare professionals; the relationship between serum VLCSFAs and CHD risk may not be generalizable to other populations. More importantly, there has been no research exploring the relationship between serum VLCSFAs and MI as well as stroke.

Our study, for the first time to our knowledge, investigated the effects of four types of VLCSFAs in serum on MI and stroke among general US adults. We observed a consistent inverse relationship between VLCSFAs and MI as well as stroke in this population. In addition, we also observed an inverse association between VLCSFAs and HF, which is consistent with the findings of the CHS cohort. Furthermore, we explored the mediation role of neutrophil‐related inflammatory markers in the association between VLCSFAs and cardiovascular outcomes because high neutrophil count has been found to be causally associated with a higher risk of cardiovascular events (Luo et al. 2023). In the mediation analysis, we found that SII, NER, NHR, and NLR may partially mediate the relationship between VLCSFAs and HF. Although the mechanisms underlying the inverse association between serum VLCSFAs and cardiovascular outcomes remain unclear, systemic inflammation may play a key role in this association because inflammation has been widely recognized as a primary driver of CVDs (Henein et al. 2022). The β‐oxidation of VLCSFAs is mainly regulated by peroxisome proliferator‐activated receptor (PPAR) δ. PPARδ has several inhibitory effects on inflammation including inhibition of NF‐κB activation, release of anti‐inflammatory corepressor B‐cell lymphoma 6 (BCL‐6) protein, and induction of anti‐inflammatory mediators such as transforming growth factor‐β (TGF‐β) (Wahli and Michalik 2012).

Several mechanisms beyond inflammation may underlie these associations. VLCSFAs are preferentially incorporated into ceramides and sphingomyelins, so that circulating VLCSFA concentrations largely mirror those of VLCSFA‐containing sphingolipids (Lemaitre and King 2022). In the Cardiovascular Health Study, sphingolipids carrying C22:0 and C24:0 were associated with lower incident heart failure, whereas palmitate‐containing species were associated with higher risk, suggesting that the balance between them, rather than total sphingolipid concentrations, may determine cardiac risk (Lemaitre et al. 2019). As the predominant acyl chains of sphingomyelin and myelin lipids, VLCSFAs also modulate membrane order, lipid‐raft formation and interleaflet interdigitation, thereby influencing receptor signaling and membrane protein function (Yeboah et al. 2021). In addition, they reflect fatty acid elongation and overall metabolic health, correlating inversely with triglycerides and de novo lipogenesis and favorably with insulin sensitivity (Ardisson et al. 2020). These mechanisms remain hypothetical pending experimental confirmation.

This study has several strengths. Firstly, the study population includes residents of multiple races in American communities, which has good representativeness. Secondly, serum VLCSFAs were measured by the US CDC using standardized methods and undergo strict quality control, ensuring the objectivity and accuracy of VLCSFAs detection. Lastly, this study examined the mediating effect of neutrophil‐related inflammatory markers in the association between VLCSFAs and HF for the first time and provided new evidence for the beneficial effects of VLCSFAs on MI and stroke at the population level. However, our study has limitations that need to be considered when interpreting the results of this study. First of all, NHANES is a cross‐sectional study and results from a cross‐sectional study cannot establish a causal relationship. Second, MI, HF, and stroke were ascertained from participants' self‐report of a physician‐made diagnosis, a method that is routinely used to estimate the prevalence of cardiovascular disease in NHANES (Lopez‐Jimenez et al. 2009). However, self‐reported diagnoses are inevitably subject to misclassification. Because the outcomes were ascertained uniformly for all participants and independently of serum VLCSFA concentrations, such misclassification is unlikely to be differential with respect to the exposure. Although non‐differential misclassification of a binary outcome is often assumed to bias the odds ratio toward the null, this assumption cannot be made with certainty and has well‐recognized exceptions in which the direction of bias is unpredictable (Yland et al. 2022). Whether our estimates are conservative or exaggerated therefore remains unknown, and this limitation should be considered when interpreting the present findings. Third, although we included multiple confounding factors, the possibility of residual confounding by unmeasured factors cannot be totally ruled out.

In conclusion, our findings suggest that VLCSFA deficiency may constitute an under‐recognized cardiovascular risk factor, and that part of this excess risk could operate through neutrophil‐related inflammatory pathways. Should these associations prove causal, maintaining adequate circulating VLCSFA concentrations—particularly of C24:0—might represent a modifiable target for cardiovascular prevention, although this requires confirmation in prospective cohort studies and randomized controlled trials.

## Author Contributions

**Xinyu Chen:** formal analysis, writing – original draft. **Long Zhou:** conceptualization, methodology, formal analysis, funding acquisition, writing – original draft. **Jun Cheng:** conceptualization, supervision, writing – review and editing.

## Funding

Dr. Long Zhou was funded by Sichuan Science and Technology Program (No. 2024YFFK0284).

## Ethics Statement

The study was approved by the National Center for Health Statistics Institutional Review Board (Protocol #2011‐17) and all participants provided written informed consent.

## Conflicts of Interest

The authors declare no conflicts of interest.

## Supporting information


**Table S1:** ORs (95% CIs) for the associations of serum very‐long‐chain saturated fatty acids (VLCSFAs) with myocardial infarction, heart failure and stroke (*N* = 4400).

## Data Availability

The data that support the findings of this study are available from the corresponding author upon reasonable request.
