# Supplementary material for: Serum Very‐Long‐Chain Saturated Fatty Acids and the Mediating Role of Systemic Immune Inflammation in Cardiovascular Disease Risk
Source: Food Sci Nutr. 2026 Nov 9;14(11):e72573. doi: 10.1002/fsn3.72573 (PMC13746176; doi:10.1002/fsn3.72573)
Supplement: Supplementary file 1 [file FSN3-14-e72573-s001.docx]

**Table S1. ORs (95% CIs) for the associations of serum very-long-chain saturated fatty acids (VLCSFAs) with myocardial infarction, heart failure and stroke (N = 4400).**

|  | **Myocardial infarction** | | |  | **Heart failure** | | |  | **Stroke** | | |
| --- | --- | --- | --- | --- | --- | --- | --- | --- | --- | --- | --- |
| **Fatty acid** | **Case No. (%)** | **Model 1** | **Model 2** |  | **Case No. (%)** | **Model 1** | **Model 2** |  | **Case No. (%)** | **Model 1** | **Model 2** |
| **C20:0 (Arachidic acid)** |  |  |  |  |  |  |  |  |  |  |  |
| T1 (lowest: <20.9) | 80 (5.5) | 1.91 (1.28-2.84), P=0.001 | 1.72 (1.14-2.60), P=0.009 |  | 67 (4.6) | 1.63 (1.09-2.43), P=0.016 | 1.44 (0.95-2.17), P=0.086 |  | 60 (4.1) | 1.29 (0.87-1.91), P=0.206 | 1.20 (0.80-1.79), P=0.376 |
| T2 (Ref.: 20.9~25.2) | 42 (2.9) | 1 (Ref.) | 1 (Ref.) |  | 44 (3.1) | 1 (Ref.) | 1 (Ref.) |  | 51 (3.5) | 1 (Ref.) | 1 (Ref.) |
| T3 (highest: ≥25.3) | 45 (3.0) | 1.12 (0.72-1.75), P=0.606 | 0.97 (0.61-1.53), P=0.881 |  | 41 (2.7) | 0.83 (0.53-1.29), P=0.400 | 0.80 (0.50-1.26), P=0.335 |  | 47 (3.1) | 0.77 (0.51-1.17), P=0.216 | 0.74 (0.48-1.13), P=0.161 |
| Continuous (per 1 μmol/L) | - | 0.96 (0.93-0.99), P=0.005 | 0.95 (0.92-0.98), P=0.003 |  | - | 0.97 (0.94-1.00), P=0.065 | 0.98 (0.95-1.01), P=0.133 |  | - | 0.98 (0.95-1.01), P=0.194 | 0.98 (0.96-1.01), P=0.260 |
| **C22:0 (Docosanoic acid)** |  |  |  |  |  |  |  |  |  |  |  |
| T1 (lowest: <59.0) | 82 (5.6) | 1.44 (0.98-2.10), P=0.062 | 1.20 (0.80-1.79), P=0.374 |  | 83 (5.7) | 2.15 (1.43-3.21), P<0.001 | 1.97 (1.30-3.00), P=0.002 |  | 70 (4.8) | 1.84 (1.21-2.78), P=0.004 | 1.75 (1.14-2.67), P=0.010 |
| T2 (Ref.: 59.0~71.9) | 48 (3.3) | 1 (Ref.) | 1 (Ref.) |  | 38 (2.6) | 1 (Ref.) | 1 (Ref.) |  | 38 (2.6) | 1 (Ref.) | 1 (Ref.) |
| T3 (highest: ≥72.0) | 37 (2.5) | 0.78 (0.50-1.23), P=0.285 | 0.69 (0.43-1.09), P=0.114 |  | 31 (2.1) | 0.75 (0.46-1.22), P=0.248 | 0.77 (0.46-1.27), P=0.299 |  | 50 (3.4) | 1.21 (0.78-1.87), P=0.391 | 1.24 (0.79-1.95), P=0.348 |
| Continuous (per 1 μmol/L) | - | 0.97 (0.96-0.99), P<0.001 | 0.98 (0.97-0.99), P<0.001 |  | - | 0.97 (0.96-0.98), P<0.001 | 0.98 (0.97-0.99), P<0.001 |  | - | 0.99 (0.98-1.00), P=0.003 | 0.99 (0.98-1.00), P=0.010 |
| **C23:0 (Tricosanoic acid)** |  |  |  |  |  |  |  |  |  |  |  |
| T1 (lowest: <25.2) | 91 (6.2) | 2.38 (1.59-3.57), P<0.001 | 2.26 (1.48-3.45), P<0.001 |  | 75 (5.1) | 1.90 (1.27-2.83), P=0.002 | 1.73 (1.14-2.62), P=0.010 |  | 66 (4.5) | 1.57 (1.05-2.34), P=0.027 | 1.52 (1.01-2.29), P=0.045 |
| T2 (Ref.: 25.2~31.3) | 37 (2.5) | 1 (Ref.) | 1 (Ref.) |  | 42 (2.8) | 1 (Ref.) | 1 (Ref.) |  | 46 (3.1) | 1 (Ref.) | 1 (Ref.) |
| T3 (highest: ≥31.4) | 39 (2.7) | 1.10 (0.69-1.76), P=0.692 | 1.13 (0.70-1.84), P=0.615 |  | 35 (2.4) | 0.73 (0.46-1.17), P=0.190 | 0.80 (0.49-1.29), P=0.360 |  | 46 (3.1) | 0.85 (0.55-1.30), P=0.441 | 0.93 (0.60-1.45), P=0.754 |
| Continuous (per 1 μmol/L) | - | 0.94 (0.92-0.97), P<0.001 | 0.95 (0.92-0.97), P<0.001 |  | - | 0.93 (0.91-0.96), P<0.001 | 0.94 (0.92-0.97), P<0.001 |  | - | 0.95 (0.93-0.98), P<0.001 | 0.96 (0.94-0.99), P=0.002 |
| **C24:0 (Lignoceric acid)** |  |  |  |  |  |  |  |  |  |  |  |
| T1 (lowest: <50.1) | 90 (6.2) | 1.62 (1.12-2.34), P=0.011 | 1.37 (0.93-2.01), P=0.109 |  | 88 (6.1) | 2.42 (1.61-3.62), P<0.001 | 2.12 (1.40-3.22), P<0.001 |  | 74 (5.1) | 1.93 (1.28-2.90), P=0.002 | 1.91 (1.25-2.90), P=0.003 |
| T2 (Ref.: 50.1~61.7) | 50 (3.4) | 1 (Ref.) | 1 (Ref.) |  | 36 (2.4) | 1 (Ref.) | 1 (Ref.) |  | 38 (2.6) | 1 (Ref.) | 1 (Ref.) |
| T3 (highest: ≥61.8) | 27 (1.8) | 0.54 (0.34-0.88), P=0.013 | 0.49 (0.30-0.81), P=0.006 |  | 28 (1.9) | 0.75 (0.45-1.24), P=0.266 | 0.81 (0.48-1.36), P=0.419 |  | 46 (3.1) | 1.17 (0.75-1.83), P=0.485 | 1.22 (0.77-1.93), P=0.396 |
| Continuous (per 1 μmol/L) | - | 0.97 (0.96-0.98), P<0.001 | 0.97 (0.96-0.98), P<0.001 |  | - | 0.96 (0.95-0.97), P<0.001 | 0.97 (0.96-0.98), P<0.001 |  | - | 0.98 (0.97-0.99), P<0.001 | 0.98 (0.97-0.99), P=0.002 |
| **Total VLCSFAs** |  |  |  |  |  |  |  |  |  |  |  |
| T1 (lowest: <157.0) | 87 (5.9) | 1.73 (1.18-2.55), P=0.005 | 1.45 (0.97-2.17), P=0.071 |  | 85 (5.8) | 2.28 (1.52-3.42), P<0.001 | 2.00 (1.31-3.05), P=0.001 |  | 72 (4.9) | 1.72 (1.15-2.56), P=0.008 | 1.69 (1.12-2.55), P=0.013 |
| T2 (Ref.: 157.0~188.9) | 44 (3.1) | 1 (Ref.) | 1 (Ref.) |  | 37 (2.6) | 1 (Ref.) | 1 (Ref.) |  | 42 (2.9) | 1 (Ref.) | 1 (Ref.) |
| T3 (highest: ≥189.0) | 36 (2.4) | 0.82 (0.52-1.29), P=0.386 | 0.71 (0.44-1.14), P=0.156 |  | 30 (2.0) | 0.72 (0.44-1.18), P=0.193 | 0.73 (0.44-1.22), P=0.229 |  | 44 (3.0) | 0.91 (0.59-1.41), P=0.676 | 0.96 (0.61-1.50), P=0.843 |
| Continuous (per 1 μmol/L) | - | 0.99 (0.98-0.99), P<0.001 | 0.99 (0.99-0.99), P<0.001 |  | - | 0.99 (0.98-0.99), P<0.001 | 0.99 (0.99-0.99), P<0.001 |  | - | 0.99 (0.99-1.00), P=0.001 | 0.99 (0.99-1.00), P=0.004 |

Model 1: adjusted for age and sex. Model 2: Model 1 plus ethnicity, education, marital status, family monthly poverty levels, smoking status, drinking status, sedentary time, healthy eating index, body mass index, hypertension, diabetes, and hypercholesterolemia. Tertile 2 was the reference group. OR, odds ratio; CI, confidence interval.
